# Investigating measurement invariance of the Emotion Regulation Questionnaire-8 (ERQ-8) across 29 countries

**DOI:** 10.1007/s12144-022-04220-6

**Published:** 2023-01-06

**Authors:** Matthias Burghart, Alexander H. J. Sahm, Daniela Mier

**Affiliations:** Department of Psychology, University of Konstanz, Konstanz, Germany

**Keywords:** Measurement invariance, Cross-cultural assessment, Affect regulation, Emotion regulation questionnaire

## Abstract

The widely used Emotion Regulation Questionnaire (ERQ) measures the habitual use of cognitive reappraisal and expressive suppression. Recently, a more economical 8-item version of the ERQ was proposed that showed good model fit. We assessed whether the latent constructs of the ERQ-8 are generalizable across different countries and cultures. To this end, we used data from the COVIDiSTRESS survey and investigated measurement invariance of the ERQ-8 in a large sample that included 11,288 individuals from 29 countries with diverse cultural backgrounds. Our analyses revealed configural and metric invariance of the ERQ-8 in 14 countries. The results suggest that emotion regulation strategies may not readily converge across all cultures. This underscores the importance of testing measurement invariance before interpreting observed differences and similarities between countries.

**Supplementary information:**

The online version contains supplementary material available at 10.1007/s12144-022-04220-6.

Emotion regulation refers to our ability to modify feelings and behavior (Gross, 1998). This includes the use of different emotion regulation strategies to either downregulate negative, or upregulate positive emotions (McRae & Gross, 2020). For instance, one might try to distract oneself to calm down when angry, or share great news with loved ones to prolong positive feelings (Gross, 2014). Numerous studies have shown that effective emotion regulation is crucial for well-being in everyday life (e.g., Kobylińska et al., 2022), and that emotion *dysregulation* is found in many disorders, such as depression (Hui et al., 2021), hypochondriasis (Bailer et al., 2017), and borderline personality disorder (Chapman, 2019).

Two extensively investigated emotion regulation strategies are cognitive reappraisal and expressive suppression (McRae & Gross, 2020). The former refers to the cognitive reinterpretation of an emotional event, thereby altering its emotional impact (e.g., telling oneself that this is just a movie and no one got hurt; McRae & Gross, 2020). According to James Gross’ Process Model of Emotion Regulation, this strategy is considered antecedent-focused, meaning that it occurs before the emotional response has fully developed (Gross & John, 2003). In contrast, suppression refers to a response-oriented strategy that involves inhibiting the associated behavioral expression of an already completely developed emotion (e.g., keeping a neutral face even though one is sad; Gross & John, 2003; McRae & Gross, 2020). While both strategies have been shown to be effective in the short-term (Germain & Kangas, 2015), studies on long-term effects suggest that the habitual use of suppression is associated with poor psychological outcomes (Low et al., 2021), whereas reappraisal is positively associated with physical health (Appleton et al., 2013), academic achievement (Ivcevic & Brackett, 2014), and resilience (Kuhlman et al., 2021). Interestingly, there is little evidence of cultural differences in reappraisal, but strong support that the negative effects of habitual suppression are culture-specific (Ramzan & Amjad, 2017; Schunk et al., 2022). Due to differences in norms and values, people in collectivistic cultures are more likely to suppress emotions to maintain social harmony than people in individualistic cultures (Matsumoto et al., 2008). Expressive suppression is therefore more common and acceptable among collectivist-oriented individuals and consequently associated with fewer or no adverse outcomes (Ramzan & Amjad, 2017). This cross-cultural variation raises questions about the comparability of emotion regulation assessment tools across many different countries.

One tool commonly used to assess individual differences in the dispositional use of emotion regulation strategies is the Emotion Regulation Questionnaire (ERQ; Gross & John, 2003). The ERQ comprises 10 items, six of which measure cognitive reappraisal and four of which measure expressive suppression. To date, the ERQ has been translated into 37 different languages (Stanford University, 2022) and has been validated across a wide range of countries, with the original 10-item two-factor structure generally showing good model fit (e.g., Balzarotti et al., 2010; D’Argembeau & Van der Linden, 2006; Matsumoto et al., 2008; Melka et al., 2011). However, these validation studies have been criticized in the past for relying exclusively on student samples (e.g., Spaapen et al., 2014). Some of the studies evaluating the ERQ in non-student samples failed to replicate the same factor structure and ended up with a 9-item (e.g., Spaapen et al., 2014) or 8-item (e.g., Balzarotti, 2021) solution. Such discrepancies in the factor structure of the ERQ may be due to differences across samples or cultures and underscore the importance of testing measurement invariance (Boer et al., 2018; Gong et al., 2021). Measurement invariance indicates whether an instrument measures the same underlying latent construct across different groups (e.g., countries) and is therefore a prerequisite for a meaningful interpretation of observed differences and similarities between these groups (Milfont & Fischer, 2010). However, few cross-cultural comparative studies examine measurement invariance before drawing conclusions from their data (Boer et al., 2018).

## The present study

The ERQ is a questionnaire used worldwide to assess the habitual use of emotion regulation strategies. Recently, the feasibility of a more economical 8-item version has been demonstrated (Balzarotti, 2021). This makes it a promising candidate for large cross-cultural studies on emotion regulation. Here, we investigate measurement invariance of the ERQ-8 in a large and diverse sample of individuals from 29 different countries to explore the validity of the 2-factor solution, and to provide norm values across different nations.

## Methods

### Sample

Data for this study was obtained from the global COVIDiSTRESS survey, which collected data on psychological and behavioral outcomes one year into the COVID-19 pandemic (Blackburn et al., 2022). The survey was conducted in 137 different countries during the summer of 2021 and yielded responses from a total of 15,740 participants, with a range of 1 to 2260 participants per country. To the best of our knowledge, there are no clear recommendations for minimum sample sizes in confirmatory factor analysis (CFA) studies (Wolf et al., 2013). Thus, we decided to include those countries with at least 100 complete responses to maximize cultural variance while maintaining adequate power for our analyses (Boer et al., 2018; Ruggeri et al., 2022). This resulted in a total sample of 11,288 individuals from 29 countries. The demographic characteristics can be found in Table 1 (for demographic information on the original COVIDiSTRESS survey sample, see Blackburn et al., 2022).

**Table 1:** Demographic characteristics of participants from the 29 included countries

Country	N	Mean age (SD)	% Students	% Women	% Higher education^a^
Brazil	388	38.1 (13.2)	13.9	72.7	96.4
Bulgaria	258	40.6 (16.7)	12.4	74.4	88.8
Colombia	434	40.4 (12.4)	7.4	67.1	95.6
Costa Rica	216	36.3 (11.0)	3.7	72.2	96.8
Czech Republic	296	33.8 (11.3)	20.9	69.6	85.1
Ecuador	206	32.7 (11.4)	18.4	67.0	96.6
Estonia	205	39.4 (10.4)	3.4	86.3	80.5
Finland	877	46.3 (10.4)	3.8	79.2	78.1
Germany	128	36.8 (13.0)	13.3	70.3	86.7
Guatemala	215	37.5 (14.4)	17.7	86.5	97.2
Honduras	305	25.4 (8.3)	52.8	66.9	84.9
Ireland	301	29.6 (11.0)	35.2	68.8	99.0
Italy	266	45.4 (16.1)	11.3	73.3	75.2
Japan	2013	45.5 (11.1)	2.4	41.3	53.7
Kyrgyzstan	188	32.9 (13.1)	25.0	83.5	69.7
Malaysia	167	27.6 (8.6)	58.1	70.7	97.0
Norway	317	40.8 (13.8)	8.5	81.7	88.0
Portugal	381	32.9 (14.6)	50.1	70.9	87.4
Russian Federation	1764	26.2 (10.7)	31.3	70.9	75.9
Slovakia	265	35.0 (13.4)	25.7	89.1	84.9
Spain	472	40.5 (13.7)	12.3	64.6	96.0
Sweden	108	38.6 (13.7)	13.0	81.5	88.9
Switzerland	522	45.2 (19.2)	10.9	63.6	70.7
Taiwan	197	35.0 (9.7)	11.2	62.9	97.0
Turkey	145	24.3 (8.6)	64.8	67.6	44.8
Uganda	119	24.3 (3.9)	71.4	61.9	99.2
Ukraine	209	31.7 (10.1)	7.7	64.6	96.2
United Kingdom	113	37.3 (13.2)	15.0	77.0	94.7
Uruguay	213	42.2 (13.1)	4.2	86.4	92.5

^a^Higher education comprises individuals who have completed or are currently completing a (post-)university degree

### Materials

The ERQ-8 does not include Item 1 (“*When I want to feel more positive emotion (such as joy or amusement), I change what I’m thinking about.*”; reappraisal) and Item 3 (“*When I want to feel less negative emotion (such as sadness or anger), I change what I’m thinking about.*”; reappraisal) of the ERQ-10. Participants were asked to rate each statement on a Likert scale from 1 (“*strongly disagree*”) to 7 (“*strongly agree*”). All items were administered in the official language of the respective country^1^.

### Procedure

Official translations of the ERQ were used in all countries. Further details on the entire COVIDiSTRESS study procedure, including inclusion criteria and data post-processing, can be found in Blackburn et al. (2022). For the purposes of our study, we obtained the open-access dataset from the Open Science Framework (https://osf.io/36tsd/; Blackburn et al., 2022). Participants who did not complete the ERQ-8 or had items missing were removed (*n* = 2842). Following listwise deletion, 29 countries with at least 100 participants were included.

### Analysis

Data was analyzed using CFA with maximum likelihood estimation in *R* with the *lavaan* package (Rosseel, 2012). However, because our data were not multivariate normal, we also performed our analyses using robust maximum likelihood estimation. This did not change the results, and is reported in the supplementary material (see Supplementary Tables 1 and 2). Our model allowed correlation of the factors reappraisal (items 5, 7, 8, and 10) and suppression (items 2, 4, 6, and 9). We followed the customary forward-step approach to measurement invariance (Sass, 2011), by (1) estimating our model in each country separately, and subsequently testing (2) a configural invariance model (i.e., factor structure held equal), (3) a metric invariance model (i.e., factor structure and factor loadings held equal), and (4) a scalar invariance model (i.e., factor structure, factor loadings, and intercepts held equal).

We assessed the fit of our country-wise and configural invariance models using various fit indices (CFI > 0.95, TLI > 0.95, RMSEA < 0.08, SRMR < 0.10; Schermelleh-Engel et al., 2003). For our model comparisons, cut-offs indicating non-invariance for comparisons between models 2 and 3 ($-0.020\ge {\Delta }\text{CFI}$, $0.030\le {\Delta }\text{RMSEA}$) and between models 3 and 4 ($-0.010\ge {\Delta }\text{CFI}$, $0.010\le {\Delta }\text{RMSEA}$; Svetina et al., 2020) were used. In addition, we computed norm values (t-scores) for each country that showed the expected factor structure.

## Results

### Confirmatory factor analysis

Model fit was acceptable in 14 of the 29 countries (Table 2). Norm values are presented in Supplementary Tables 3 and 4 for the 14 countries in which the expected factor structure of the ERQ-8 was met. Factor correlations and internal consistencies are reported in Supplementary Table 5.

**Table 2:** Model fit of the two-factor ERQ-8 model across countries

Country	${\varvec{\chi }}^{2}$	*p* (${\varvec{\upchi }}^{2}$)	CFI	TLI	RMSEA	SRMR
Brazil*	52.4	< 0.001	0.969	0.954	0.067	0.052
Bulgaria*	44.2	0.001	0.968	0.952	0.071	0.049
Colombia*	42.6	0.001	0.985	0.977	0.053	0.043
Costa Rica*	22.3	0.269	0.995	0.993	0.028	0.042
Czech Republic	64.3	< 0.001	0.952	0.929	0.090	0.072
Ecuador	41.1	0.002	0.952	0.929	0.075	0.066
Estonia	54.5	< 0.001	0.957	0.936	0.096	0.091
Finland	128.8	< 0.001	0.953	0.931	0.081	0.059
Germany*	22.9	0.241	0.987	0.981	0.040	0.047
Guatemala	55.2	< 0.001	0.921	0.884	0.094	0.076
Honduras	61.1	< 0.001	0.933	0.902	0.085	0.066
Ireland*	51.2	< 0.001	0.974	0.962	0.075	0.059
Italy	60.1	< 0.001	0.954	0.932	0.090	0.083
Japan	559.2	< 0.001	0.895	0.845	0.119	0.093
Kyrgyzstan	63.8	< 0.001	0.873	0.813	0.112	0.091
Malaysia*	24.0	0.196	0.990	0.985	0.040	0.048
Norway*	54.1	< 0.001	0.968	0.952	0.076	0.072
Portugal*	52.3	< 0.001	0.977	0.966	0.068	0.051
Russian Federation	403.4	< 0.001	0.901	0.853	0.107	0.081
Slovakia*	42.7	0.001	0.979	0.969	0.069	0.060
Spain*	34.2	0.018	0.991	0.987	0.041	0.030
Sweden	46.8	< 0.001	0.932	0.900	0.117	0.081
Switzerland*	47.0	< 0.001	0.976	0.965	0.053	0.047
Taiwan	93.3	< 0.001	0.771	0.662	0.141	0.095
Turkey*	31.6	0.035	0.973	0.961	0.068	0.051
Uganda	54.5	< 0.001	0.855	0.786	0.125	0.102
Ukraine	47.1	< 0.001	0.952	0.929	0.084	0.077
United Kingdom*	16.7	0.610	1.00	1.01	0.000	0.043
Uruguay	55.8	< 0.001	0.936	0.906	0.095	0.070

The asterisk (*) indicates countries with acceptable model fit. Degrees of freedom = 19

### Multi group confirmatory factor analysis and measurement invariance

We performed stepwise measurement invariance analyses with both the full sample (*N* = 29) and the subsample of countries that showed acceptable fit in the CFA (*N* = 14). When analyzing the smaller sample, we found acceptable fit indices for the configural invariance model as well as acceptable fit differences for the metric invariance model, but not when analyzing the full sample (Table 3).

**Table 3:** Fit indices for different measurement invariance models

Model	${\upchi }^{2}$	$\mathrm{df}$	${\Delta }{\upchi }^{2}$	$\Delta\mathrm{df}$	$\mathrm{CFI}$	$\Delta\mathrm{CFI}$	$\mathrm{RMSEA}$	$\Delta\mathrm{RMSEA}$	$\mathrm{SRMR}$	$\Delta\mathrm{SRMR}$
29 Countries										
CI	2327	551			0.944		0.091		0.063	
MI	2882	719	555*	168	0.931	− 0.012	0.088	− 0.003	0.077	0.014
SI	4732	887	1850*	168	0.878	− 0.053	0.106	0.018	0.095	0.018
14 Countries										
CI	538	266			0.980		0.059		0.044	
MI	736	344	198*	78	0.971	− 0.009	0.062	0.003	0.060	0.016
SI	1188	422	452*	78	0.943	− 0.028	0.079	0.016	0.074	0.014

CI = Configural Invariance; MI = Metric Invariance; SI = Scalar Invariance

*Statistically significant at *p* < 0.05

## Discussion

We assessed the suitability of the ERQ-8 for cross-cultural comparisons, by investigating its measurement invariance, and provided norm values for 14 countries. Although measurement invariance is a prerequisite for meaningful group comparisons (Milfont & Fischer, 2010), only a fraction of cross-cultural research establishes it before generalizing their findings across different samples (Boer et al., 2018). Our results emphasize the importance of such an approach, as the original factor structure of the ERQ-8 could only be replicated in 14 of the 29 countries (i.e., Brazil, Bulgaria, Colombia, Costa-Rica, Germany, Ireland, Malaysia, Norway, Portugal, Slovakia, Spain, Switzerland, Turkey, and the UK). Moreover, while metric invariance was achieved in the same group of countries, allowing comparisons of correlation coefficients; scalar invariance was not given, i.e., mean comparisons are *not* adequate.

In view of these findings, the question arises as to why measurement invariance was not observed in all 29 countries. We suggest two possible explanations. The original ERQ might have performed better than the shorter ERQ-8 because this more parsimonious version may be flawed by the absence of two items. However, we consider this unlikely, as previous research has also demonstrated poor fit of the original ERQ in non-student samples (Balzarotti, 2021; Spaapen et al., 2014). Another reason may be that the latent constructs measured by the ERQ-8 cannot be readily generalized across all countries and cultures. The ERQ was developed by Gross and John (2003) on a student sample from a so-called Western, Educated, Industrialized, Rich, and Democratic (WEIRD) country. It is commonly recognized that people from WEIRD nations differ fundamentally from people from non-WEIRD nations in many psychological processes (Cheon et al., 2020). This is not to say that the ERQ/ERQ-8 should not be used in non-WEIRD countries, but rather that comparisons across countries without measurement invariance should be made with caution, as they may be measuring conceptually different constructs (Boer et al., 2018). Future research should aim to shed more light on this issue and strive to include more samples from non-WEIRD countries, particularly since differences in the use of suppression and reappraisal have already been linked to different cultural values (Matsumoto et al., 2008; Ramzan & Amjad, 2017; Schunk et al., 2022).

In contrast to Balzarotti (2021), we did not find a good model fit of the ERQ-8 among Italians. However, a closer inspection of the sample composition shows that our sample, although similar in age, includes more female participants (52% vs. 73.3%). This may explain the differences in model fit, but would also suggest that the latent constructs measured by the ERQ-8 vary between men and women. However, this can be doubted, because studies from different cultures have found acceptable measurement invariance of the ERQ across genders (Melka et al., 2011; Zhang & Bian, 2020). Further studies are needed to identify causes of measurement non-invariance that go beyond cultural factors.

### Limitations

Despite the strengths of our study, such as the large number of participants from 29 countries with diverse cultural backgrounds, our results must be interpreted with some limitations in mind. First, the samples might not be representative of their respective country’s true population. Although the number of students is rather small, the overall educational level is high. That is, people of lower socioeconomic status are likely not fully represented in this study. This must also be taken into account when using the norm values from our supplementary materials. Second, the COVIDiSTRESS survey was carried out in the context of the COVID-19 pandemic. This may have raised participants’ stress levels and influenced their responses, possibly resulting in fewer functional emotion regulation strategies being reported. Third, the German translation of the ERQ-8 (which was used in Germany and Switzerland) lacked the negation of item 9. Although this was easily resolved in our analyses (by reverse coding the responses), from a psychometric point of view this item no longer corresponds to the original item (Schriesheim et al., 1991). Fourth, the internal consistency of both subscales was poor in some countries (*α* < 0.70), which may have possibly influenced our model fit despite being due to reliability issues and not due to actual differences in the latent constructs measured. Finally, based on our data, we cannot draw firm conclusions about the reasons for the differences in measurement invariance across countries. We have discussed potential explanations, but these remain to be explored in future studies.

### Conclusion

The ERQ-8 is an economical instrument for assessing the habitual use of suppression and reappraisal. It shows configural and metric invariance across 14 different countries, allowing cross-cultural comparisons and generalizability of correlation coefficients. However, the lack of measurement invariance in 15 additional countries suggests possible differences in latent constructs measured by the ERQ-8. This highlights the need to test measurement invariance in future cross-cultural studies.

## Supplementary information

Below is the link to the electronic supplementary material.

ESM 1 (DOCX 57.6 KB)

## Footnotes

1 Item 9 was reversed prior to all analyses in the German and Swiss samples because the German translation used in the COVIDiSTRESS survey lacked the negation that is used in the original English version.
