# Supplementary material for: Investigating measurement invariance of the Emotion Regulation Questionnaire-8 (ERQ-8) across 29 countries
Source: Curr Psychol. 2023 Jan 6:1–7. Online ahead of print. doi: 10.1007/s12144-022-04220-6 (PMC9817443; doi:10.1007/s12144-022-04220-6)
Supplement: Supplementary file 1 — (DOCX 57.6 KB) [file 12144_2022_4220_MOESM1_ESM.docx]

**Supplementary Materials**

**Investigating Measurement Invariance of the Emotion Regulation Questionnaire-8
(ERQ-8) Across 29 Countries**

Matthias Burghart, Alexander H. J. Sahm, Daniela Mier

University of Konstanz, Germany

**Supplementary Table 1.** *Model Fit of the Two-Factor ERQ-8 Model Across Countries (Using Robust Maximum Likelihood Estimation)*

| **Country** | $\boldsymbol{\chi}^{\mathbf{2}}$ | **CFI** | **TLI** | **RMSEA** | **SRMR** |
| --- | --- | --- | --- | --- | --- |
| Brazil* | 52.4 | .972 | .958 | .064 | .052 |
| Bulgaria* | 44.2 | .973 | .960 | .065 | .049 |
| Colombia* | 42.6 | .986 | .979 | .051 | .043 |
| Costa Rica* | 22.3 | 1.000 | 1.000 | .006 | .042 |
| Czech Republic | 64.3 | .957 | .937 | .084 | .072 |
| Ecuador | 41.1 | .956 | .935 | .071 | .066 |
| Estonia | 54.5 | .961 | .943 | .090 | .091 |
| Finland | 128.8 | .955 | .933 | .080 | .059 |
| Germany* | 22.9 | .992 | .989 | .031 | .047 |
| Guatemala | 55.2 | .929 | .895 | .088 | .076 |
| Honduras | 61.1 | .940 | .912 | .080 | .066 |
| Ireland* | 51.2 | .976 | .964 | .072 | .059 |
| Italy | 60.1 | .957 | .937 | .086 | .083 |
| Japan | 559.2 | .896 | .847 | .118 | .093 |
| Kyrgyzstan | 63.8 | .872 | .811 | .110 | .091 |
| Malaysia* | 24.0 | .997 | .996 | .020 | .048 |
| Norway* | 54.1 | .970 | .955 | .074 | .072 |
| Portugal* | 52.3 | .981 | .971 | .062 | .051 |
| Russian Federation | 403.4 | .902 | .856 | .106 | .081 |
| Slovakia* | 42.7 | .980 | .970 | .066 | .060 |
| Spain* | 34.2 | .992 | .989 | .038 | .030 |
| Sweden | 46.8 | .926 | .892 | .121 | .081 |
| Switzerland* | 47.0 | .978 | .968 | .051 | .047 |
| Taiwan | 93.3 | .757 | .641 | .142 | .095 |
| Turkey* | 31.6 | .970 | .956 | .071 | .051 |
| Uganda | 54.5 | .870 | .808 | .115 | .102 |
| Ukraine | 47.1 | .956 | .935 | .080 | .077 |
| United Kingdom* | 16.7 | 1.000 | 1.018 | .000 | .043 |
| Uruguay | 55.8 | .939 | .909 | .093 | .070 |

*Note*. The asterisk (*) indicates countries with acceptable model fit. Degrees of freedom = 19.

**Supplementary Table 2.** *Fit Indices for Different Measurement Invariance Models (Using Robust Maximum Likelihood Estimation)*

| **Model** | $\boldsymbol{\chi}^{\mathbf{2}}$ | $\mathbf{df}$ | $\boldsymbol{\Delta}\boldsymbol{\chi}^{\mathbf{2}}$ | $\boldsymbol{\Delta df}$ | $\mathbf{CFI}$ | $\boldsymbol{\Delta CFI}$ | $\mathbf{RMSEA}$ | $\boldsymbol{\Delta RMSEA}$ | $\mathbf{SRMR}$ | $\boldsymbol{\Delta SRMR}$ |
| --- | --- | --- | --- | --- | --- | --- | --- | --- | --- | --- |
| **29 Countries** | |  |  |  |  |  |  |  |  |  |
| CI | 2327 | 551 |  |  | .946 |  | .089 |  | .063 |  |
| MI | 2882 | 719 | 555* | 168 | .935 | -.011 | .085 | -.004 | .077 | .014 |
| SI | 4732 | 887 | 1850* | 168 | .881 | -.054 | .104 | .019 | .095 | .018 |
| **14 Countries** | |  |  |  |  |  |  |  |  |  |
| CI | 538 | 266 |  |  | .982 |  | .055 |  | .044 |  |
| MI | 736 | 344 | 198* | 78 | .975 | -.007 | .058 | .003 | .060 | .016 |
| SI | 1188 | 422 | 452* | 78 | .947 | -.028 | .076 | .018 | .074 | .014 |

*Note*. CI = Configural Invariance; MI = Metric Invariance; SI = Scalar Invariance.

^*^ Statistically significant at *p* < .05.

**Supplementary Table 3.** *Population Based Norms (T Scores) for the Reappraisal Scale of the ERQ-8 Across Countries*

| Raw Score | Brazil | Bulgaria | Colombia | Costa Rica | Germany | Ireland | Malaysia | Norway | Portugal | Slovakia | Spain | Switzer-land | Turkey | United Kingdom |
| --- | --- | --- | --- | --- | --- | --- | --- | --- | --- | --- | --- | --- | --- | --- |
| $1.00$ | 19 | 17 | 19 |  | 19 | 21 |  | 18 | 20 | 20 | 20 |  | 15 |  |
| $1.25$ |  |  |  |  |  | 23 | 17 | 20 | 22 |  | 22 | 17 |  |  |
| $1.50$ | 23 | 21 | 23 | 21 | 23 | 25 |  | 22 | 24 |  |  | 19 |  |  |
| $1.75$ | 25 | 23 | 25 | 23 |  | 27 |  | 24 | 26 | 26 | 26 | 22 |  | 27 |
| $2.00$ | 27 | 25 | 27 | 25 |  | 29 |  | 26 | 28 | 28 | 28 | 24 |  | 29 |
| $2.25$ | 29 | 27 | 29 | 26 | 30 | 31 |  | 28 | 30 | 30 | 30 | 27 | 25 | 31 |
| $2.50$ | 31 | 29 | 31 | 28 | 32 | 32 | 27 | 31 | 32 | 32 | 32 | 29 | 27 | 33 |
| $2.75$ | 33 | 31 | 32 | 30 | 34 | 34 | 29 | 33 | 34 | 34 | 34 | 31 | 29 | 35 |
| $3.00$ | 35 | 33 | 34 | 32 | 36 | 36 | 31 | 35 | 36 | 36 | 36 | 34 | 31 | 37 |
| $3.25$ | 37 | 35 | 36 | 34 | 38 | 38 | 33 | 37 | 38 | 38 | 38 | 36 | 33 | 39 |
| $3.50$ | 39 | 37 | 38 | 36 | 40 | 40 | 35 | 39 | 40 | 40 | 40 | 39 | 35 | 41 |
| $3.75$ | 41 | 39 | 40 | 38 | 42 | 42 | 38 | 41 | 42 | 41 | 42 | 41 | 38 | 43 |
| $4.00$ | 43 | 41 | 42 | 40 | 44 | 44 | 40 | 43 | 44 | 43 | 43 | 43 | 40 | 45 |
| $4.25$ | 45 | 43 | 43 | 42 | 47 | 46 | 42 | 45 | 46 | 45 | 45 | 46 | 42 | 47 |
| $4.50$ | 47 | 45 | 45 | 44 | 49 | 48 | 44 | 48 | 48 | 47 | 47 | 48 | 44 | 49 |
| $4.75$ | 49 | 47 | 47 | 45 | 51 | 50 | 46 | 50 | 50 | 49 | 49 | 50 | 46 | 51 |
| $5.00$ | 51 | 49 | 49 | 47 | 53 | 52 | 48 | 52 | 52 | 51 | 51 | 53 | 48 | 53 |
| $5.25$ | 53 | 51 | 51 | 49 | 55 | 54 | 50 | 54 | 54 | 53 | 53 | 55 | 50 | 55 |
| $5.50$ | 55 | 53 | 53 | 51 | 57 | 56 | 52 | 56 | 56 | 55 | 55 | 58 | 52 | 57 |
| $5.75$ | 57 | 55 | 55 | 53 | 59 | 57 | 54 | 58 | 58 | 57 | 57 | 60 | 54 | 59 |
| $6.00$ | 59 | 57 | 56 | 55 | 61 | 59 | 57 | 60 | 60 | 59 | 59 | 62 | 56 | 61 |
| $6.25$ | 61 | 59 | 58 | 57 | 63 | 61 | 59 | 62 | 62 | 61 | 61 | 65 | 58 | 63 |
| $6.50$ | 63 | 61 | 60 | 59 | 65 | 63 | 61 | 64 | 64 | 63 | 63 | 67 | 60 | 65 |
| $6.75$ | 65 | 63 | 62 | 61 | 68 | 65 | 63 | 67 | 66 | 65 | 65 | 70 | 63 | 67 |
| $7.00$ | 67 | 66 | 64 | 63 | 70 | 67 | 65 | 69 | 68 | 67 | 66 | 72 | 65 | 69 |
| *N* | 388 | 258 | 434 | 216 | 128 | 301 | 167 | 317 | 381 | 265 | 472 | 522 | 145 | 113 |

**Supplementary Table 4.** Population Based Norms (T Scores) for the Suppression Scale of the ERQ-8 Across Countries

| Raw Score | Brazil | Bulgaria | Colombia | Costa Rica | Germany | Ireland | Malaysia | Norway | Portugal | Slovakia | Spain | Switzer-land | Turkey | United Kingdom |
| --- | --- | --- | --- | --- | --- | --- | --- | --- | --- | --- | --- | --- | --- | --- |
| 1.00 | 31 | 31 | 33 | 35 | 27 | 31 |  | 34 | 30 | 29 | 32 | 29 | 32 |  |
| 1.25 | 33 | 33 | 35 | 36 |  | 33 | 29 | 36 | 32 | 31 | 34 | 31 | 34 | 33 |
| 1.50 | 35 | 34 | 36 | 38 | 32 | 35 | 30 | 38 | 34 | 33 | 36 | 33 | 35 | 35 |
| 1.75 | 37 | 36 | 38 | 39 | 34 | 36 | 32 | 39 | 35 | 35 | 37 | 35 | 37 | 37 |
| 2.00 | 38 | 38 | 40 | 41 | 36 | 38 | 34 | 41 | 37 | 37 | 39 | 38 | 39 | 39 |
| 2.25 | 40 | 39 | 41 | 43 | 39 | 40 | 36 | 43 | 39 | 39 | 41 | 40 | 40 | 40 |
| 2.50 | 42 | 41 | 43 | 44 | 41 | 41 | 37 | 45 | 41 | 41 | 43 | 42 | 42 | 42 |
| 2.75 | 44 | 43 | 44 | 46 | 43 | 43 | 39 | 47 | 43 | 43 | 44 | 44 | 43 | 44 |
| 3.00 | 46 | 45 | 46 | 47 | 46 | 45 | 41 | 49 | 44 | 45 | 46 | 46 | 45 | 46 |
| 3.25 | 48 | 46 | 48 | 49 | 48 | 46 | 43 | 51 | 46 | 47 | 48 | 49 | 47 | 47 |
| 3.50 | 50 | 48 | 49 | 51 | 50 | 48 | 44 | 53 | 48 | 49 | 49 | 51 | 48 | 49 |
| 3.75 | 52 | 50 | 51 | 52 | 53 | 50 | 46 | 55 | 50 | 51 | 51 | 53 | 50 | 51 |
| 4.00 | 54 | 51 | 52 | 54 | 55 | 51 | 48 | 56 | 51 | 53 | 53 | 55 | 52 | 53 |
| 4.25 | 56 | 53 | 54 | 55 | 57 | 53 | 50 | 58 | 53 | 55 | 55 | 58 | 53 | 54 |
| 4.50 | 58 | 55 | 55 | 57 | 60 | 55 | 51 | 60 | 55 | 57 | 56 | 60 | 55 | 56 |
| 4.75 | 59 | 56 | 57 | 59 | 62 | 56 | 53 | 62 | 57 | 59 | 58 | 62 | 56 | 58 |
| 5.00 | 61 | 58 | 59 | 60 | 64 | 58 | 55 | 64 | 59 | 61 | 60 | 64 | 58 | 60 |
| 5.25 | 63 | 60 | 60 | 62 | 67 | 60 | 57 | 66 | 60 | 63 | 61 | 67 | 60 | 61 |
| 5.50 | 65 | 62 | 62 | 64 | 69 | 61 | 58 | 68 | 62 | 65 | 63 | 69 | 61 | 63 |
| 5.75 | 67 | 63 | 63 | 65 | 71 | 63 | 60 | 70 | 64 | 67 | 65 | 71 | 63 | 65 |
| 6.00 | 69 | 65 | 65 | 67 |  | 65 | 62 | 72 | 66 | 69 | 67 | 73 |  | 67 |
| 6.25 | 71 | 67 | 67 | 68 |  | 66 | 64 | 73 | 67 | 71 | 68 | 75 | 66 | 68 |
| 6.50 | 73 | 68 | 68 | 70 |  | 68 | 65 | 75 | 69 | 73 | 70 | 78 | 68 | 70 |
| 6.75 | 75 | 70 | 70 | 72 |  | 70 |  | 77 | 71 | 75 | 72 |  | 69 |  |
| 7.00 | 77 | 72 | 71 | 73 |  | 71 | 69 | 79 | 73 | 77 | 73 | 82 | 71 | 74 |
| *N* | 388 | 258 | 434 | 216 | 128 | 301 | 167 | 317 | 381 | 265 | 472 | 522 | 145 | 113 |

**Supplementary Table 5.** *Factor Correlations and Internal Consistencies for Each Country*

| **Country** | ***r*** | ***p*** | ***α*_Reappraisal_** | ***α*_Suppression_** |
| --- | --- | --- | --- | --- |
| Brazil | -.028 | .648 | .81 | .73 |
| Bulgaria | .092 | .205 | .81 | .80 |
| Colombia | -.104 | .068 | .82 | .82 |
| Costa Rica | -.048 | .535 | .82 | .81 |
| Czech Republic | -.061 | .382 | .83 | .77 |
| Ecuador | -.043 | .621 | .77 | .73 |
| Estonia | .102 | .212 | .87 | .79 |
| Finland | -.013 | .753 | .79 | .79 |
| Germany | -.063 | .558 | .82 | .57 |
| Guatemala | -.191 | .041 | .76 | .74 |
| Honduras | .112 | .125 | .76 | .70 |
| Ireland | -.123 | .065 | .86 | .83 |
| Italy | .135 | .071 | .81 | .82 |
| Japan | .468 | <.001 | .81 | .69 |
| Kyrgyzstan | .400 | .002 | .65 | .74 |
| Malaysia | .070 | .443 | .82 | .78 |
| Norway | -.102 | .127 | .83 | .79 |
| Portugal | .061 | .302 | .85 | .81 |
| Russian Federation | .457 | <.001 | .74 | .71 |
| Slovakia | .074 | .288 | .89 | .76 |
| Spain | .035 | .512 | .84 | .81 |
| Sweden | -.185 | .116 | .84 | .81 |
| Switzerland | -.003 | .956 | .76 | .71 |
| Taiwan | .390 | .004 | .60 | .62 |
| Turkey | .269 | .020 | .83 | .80 |
| Uganda | .443 | .011 | .74 | .64 |
| Ukraine | .211 | .019 | .82 | .79 |
| United Kingdom | -.093 | .388 | .86 | .84 |
| Uruguay | -.084 | .325 | .78 | .74 |

*Note*. *α* = Cronbach’s alpha.
